# Дисгликемия при COVID-19 и сахарном диабете 2 типа: особенности гликемического профиля у госпитализированных пациентов и роль стероид-индуцированных нарушений

**DOI:** 10.14341/probl12840

**Published:** 2022-04-30

**Authors:** Л. Г. Стронгин, Т. А. Некрасова, Д. В. Беликина, К. Г. Корнева, А. В. Петров

**Affiliations:** Приволжский исследовательский медицинский университет; Приволжский исследовательский медицинский университет; Приволжский исследовательский медицинский университет; Приволжский исследовательский медицинский университет; Приволжский исследовательский медицинский университет

**Keywords:** непрерывное мониторирование глюкозы, COVID-19, сахарный диабет 2 типа, гипергликемия, гипогликемия, глюкокортикостероиды

## Abstract

**ОБОСНОВАНИЕ:**

ОБОСНОВАНИЕ. В литературе недостаточно данных относительно подтвержденных результатами непрерывного ­мониторирования глюкозы (НМГ) особенностей дисгликемии у госпитализированных больных COVID-19 ссопутствующим сахарным диабетом 2 типа (СД2).

**ЦЕЛЬ:**

ЦЕЛЬ. Изучить особенности гликемического профиля госпитализированных больных COVID-19 и сопутствующим СД2 по данным НМГ, оценить значение стероидной терапии в генезе дисгликемии.

**МАТЕРИАЛЫ И МЕТОДЫ:**

МАТЕРИАЛЫ И МЕТОДЫ. Обследован 21 пациент с COVID-19 и СД2 (основная группа), а также 21 пациент с СД2 без COVID-19 (контрольная группа) с помощью профессионального 4–7-дневного НМГ. Также сравнили две подгруппы больных с COVID-19 и СД2: 1) пациенты, получавшие системные глюкокортикостероиды (ГКС) во время проведения НМГ и 2) пациенты, которым НМГ проводилось после отмены ГКС.

**РЕЗУЛЬТАТЫ:**

РЕЗУЛЬТАТЫ. В группе больных COVID-19и СД2 по сравнению с контролем отмечался меньший процент времени гликемии в целевом диапазоне (32,7±20,40vs48,0±15,60%; р=0,026), были повышены показатели средней гликемии (p<0,05), но не различались доли больных с эпизодами гипогликемий (33,3% vs 38,1%; р=0,75). При этом больные, получавшие дексаметазон во время НМГ, характеризовались более высокой гипергликемией и отсутствием эпизодов гипогликемий. У больных, которым НМГ проводилась после отмены дексаметазона, гипергликемия была менее выраженной, но у 60% из них выявлялись эпизоды гипогликемии, часто — ночные, клинически значимые и не выявленные рутинными методами.

**ЗАКЛЮЧЕНИЕ:**

ЗАКЛЮЧЕНИЕ. Больные COVID-19 и СД2 отличаются выраженнойустойчивой гипергликемией, однако третьиз нихимеютэпизоды гипогликемии. Во время терапии дексаметазоном отмечается наиболее выраженная гипергликемия, безэпизодов гипогликемии. У больных, которым НМГ проводилось после отмены дексаметазона, гипергликемия менее выражена, но у 60% из них выявляются эпизоды гипогликемии, часто — ночные, клинически значимые и не диагностированные рутинными методами.Было бы целесообразно рекомендовать как минимум 5–6-кратное исследование уровня глюкозы крови (с обязательной ее оценкой в ночное время) даже стабильным больным с сочетанной патологиейпосле окончания лечения ГКС.

## Abstract (en)

**BACKGROUND:**

BACKGROUND: There is a lack of data on the features of dysglycemia in hospitalized patients with COVID-19 and concomitant diabetes mellitus (DM) confirmed by continuous glucose monitoring (CGM).

**AIM:**

AIM: to study the glycemic profile in hospitalized patients with COVID-19 and type 2 diabetes mellitus by continuous glucose monitoring and the role of steroid therapy in dysglycemiadevelopment.

**MATERIALS AND METHODS:**

MATERIALS AND METHODS: We examined 21 patients with COVID-19 and DM 2 and 21 patients with DM 2 without COVID-19 (control group) using a professional 4–7-day CGM. We also compared two subgroups of patients with COVID-19 and DM 2: 1) patients received systemic glucocorticosteroids (GCS) during CGM and 2) patients in whomCGMwas performed after discontinuation of GCS.

**RESULTS:**

RESULTS: Compared with controls, patients with COVID-19 and DM2 had lesser values of glycemic «time in range» (32.7 ± 20.40 vs 48.0 ± 15.60%, p = 0.026) andhigher parameters of mean glycemia (p <0.05) but similar proportion of patients with episodes of hypoglycemia (33.3% vs 38.1%, p = 0.75). Patients who received dexamethasone during CGM were characterized by higher hyperglycemia and the absence of episodes of hypoglycemia. In patients who hadCGM after dexamethasone discontinuation, hyperglycemia was less pronounced, but 60% of them had episodes of hypoglycemia, often nocturnal, clinically significant and not detected by routine methods.

**CONCLUSION:**

CONCLUSION: Patients with COVID-19 and DM 2had severe and persistent hyperglycemia but a third of them hadalso episodes of hypoglycemia. During therapy with dexamethasone, they had the most pronounced hyperglycemia without episodes of hypoglycemia. In patients who underwent CGM after discontinuation of dexamethasone, hyperglycemia was less pronounced but 60% of them have episodes of hypoglycemia, often nocturnal, clinically significant and not diagnosed by routine methods. It would be advisable to recommend at least a 5–6-fold study of the blood glucose level (with its obligatory assessment at night) even for stable patients with COVID-19 and DM 2after the end of GCS treatment.

## ОБОСНОВАНИЕ

Сахарный диабет (СД) считается одним из самых частых и клинически значимых коморбидных состояний у больных COVID-19, среди которых он выявляется в 15% случаев [1] и ассоциируется с более чем двукратным увеличением риска тяжелого течения болезни [2][3].

Развитие COVID-19 на фоне СД ведет к нарастанию выраженности дисгликемии, имеющей самостоятельное негативное влияние на прогноз [3–8], что справедливо в отношении как гипер [4][6][7], так и гипогликемии [5][8][9].

Усугубление гипергликемии при сочетанной патологии объясняют комплексом факторов [10–13], включая изначально худший метаболический статус с хроническим нарушением углеводного обмена, ожирением и связанным с ними низкоинтенсивным воспалением, увеличение инсулинорезистентности из-за активации воспаления при инфицировании, непосредственное повреждение β-клеток вирусом SARS-CoV2. В свою очередь, гипергликемия ведет к ухудшению иммунной защиты от инфекций [14], сопряжена с недостаточной эффективностью патогенетической терапии COVID-19 (в том числе, биологической) [15], ассоциируется с рисками электролитных нарушений, дегидратации и гиперосмолярных состояний [3], что суммарно увеличивает ее негативное влияние на прогноз.

Гипогликемия потенциально не менее опасна, особенно с учетом обусловленного ею повышения активности катехоламинов с увеличением риска аритмий и миокардиальных повреждений [3], и так характерных для COVID-19 [16–18]. Условия, способствующие возникновению гипогликемии, достаточно часто складываются при ведении больных с сочетанием COVID-19 и СД: с одной стороны, пациентам нередко требуется интенсификация инсулинотерапии, с другой — возможны нарушения режима питания, особенно в тяжелых случаях. Однако в настоящее время мало исследований, где оценивались бы частота, последствия и провоцирующие факторы гипогликемии в условиях COVID-19 в реальной клинической практике.

Особой ситуацией являются инициация, проведение и окончание терапии глюкокортикостероидами (ГКС), что подразумевает колебания степени инсулинорезистентности и дополнительный риск усугубления дисгликемии, в первую очередь стероид-индуцированной гипергликемии. Сейчас разрабатываются подходы к ее профилактике и лечению при COVID-19, однако имеющиеся инструкции в значительной степени базируются на экспертном мнении специалистов; при этом недостаток посвященных данной проблеме научных исследований подчеркивается, в том числе, авторами упомянутых рекомендаций [19].

Очевидно, что текущая клиническая ситуация требует дальнейшего изучения распространенности, характерных особенностей и триггеров нарастания дисгликемии у больных с сочетанием COVID-19 и СД, в том числе при лечении системными ГКС.

Поставленная задача предполагает тщательный и длительный контроль уровня глюкозы в биологических жидкостях, для чего может быть применена технология непрерывного мониторирования гликемии (НМГ). Отметим, что в условиях пандемии технологии НМГ оказались очень востребованы, но больше в плане удаленной оценки гликемии при СД (что важно при карантинных ограничениях) либо в качестве альтернативы традиционным частым заборам крови у тяжелых больных COVID-19 в отделении интенсивной терапии (ОРИТ) [20–25].

В то же время не хватает подтвержденных результатами НМГ научных данных относительно особенностей дисгликемии у госпитализированных больных COVID-19 и сопутствующим СД, которые получают стандартную терапию (в том числе, ГКС) в условиях общего отделения инфекционного стационара.

## ЦЕЛЬ ИССЛЕДОВАНИЯ

С учетом указанных предпосылок, была определена цель настоящего исследования: изучить особенности гликемического профиля у госпитализированных больных с COVID-19 и сопутствующим сахарным диабетом 2 типа (СД2) по данным НМГи значение стероидной терапии в генезе дисгликемии.

## МАТЕРИАЛЫ И МЕТОДЫ

## Место и время проведения исследования

Место проведения. Государственное бюджетное учреждение здравоохранения Нижегородской области«Городская клиническая больница №13 Автозаводского района города Нижнего Новгорода».

Время исследования. Набор пациентов осуществлялся в период с июня 2020г. по февраль 2021 г.

## Изучаемые популяции (одна или несколько)

В исследовании участвовали две популяции больных: 1) больные с COVID-19 и сопутствующим СД2 типа (основная группа) и 2) больные с СД2 типа без COVID-19 (группа сравнения).

Основная группа. Критерии включения в основную группу: 1) наличие COVID-19, подтвержденного результатами ПЦР, 2) госпитализация в общее отделение на базе многопрофильного стационара, перепрофилированного в инфекционный госпиталь для лечения больных COVID-19, 3) наличие сопутствующегоСД2 типа. Критерием исключения был отказ от участия в исследовании и/или от проведения НМГ.

Группа сравнения. Критерии включения в контрольную группу: 1) наличие СД2, 2) госпитализация в эндокринологическое отделение многопрофильного стационара для подбора оптимальной сахароснижающей терапии. Критерием исключения был отказ от участия в исследовании и/или от проведения НМГ.

## Способ формирования выборки из изучаемой популяции (или нескольких выборок из нескольких изучаемых популяций)

Основная группа формировалась путем сплошного включения наблюдений: в нее последовательно попадали все больные с COVID-19 и СД2, получавшие стационарное лечение в перепрофилированном в инфекционный стационар эндокринологическом отделении, которые соответствовали критериям включения и не имели критериев исключения из исследования.

Контрольная группа была сформирована методом подбора пар к наблюдениям первой выборки. Она была подобрана из числа больных СД2 типа, проходивших НМГ в условиях того же отделения и с применением того же оборудования, но вне периода его перепрофилирования в инфекционный стационар. При формировании пар учитывались пол, возрастная декада и округленный до единиц уровень гликированного гемоглобина (HbA1c).

## Дизайн исследования

Проведено одноцентровое открытое динамическое проспективное сравнительное двухвыборочное исследование, в ходе которого сравнивались результаты НМГ пациентов с СД2, имевших и не имевших COVID-19.Кроме того, на втором этапе работы сравнили две подгруппы больных с COVID-19 и СД2: 1) пациенты, получавшие системные ГКС во время проведения НМГ и 2) пациенты, которым НМГ проводилось после отмены ГКС.

## Описание медицинского вмешательства (для интервенционных исследований)

Всем больным выполнили профессиональное 4–7-дневное НМГ. Применявшаяся технология не позволяла оценить уровень глюкозы в режиме реального времени и провести дополнительные коррекционные мероприятия, но давала возможность ретроспективно выявить нарушения гликемического контроля в условиях рутинной терапии. НМГ стартовало на 1–18-й день пребывания в стационаре (в среднем 7,5±7,30 суток госпитализации).

## Методы

При определении критериев включения диагноз COVID-19 ставился на основании: 1) положительных результатов ПЦР, 2) наличия вирусного пневмонита клинически и по результатам компьютерной томографии (КТ). Диагноз СД2 у госпитализированных пациентов с COVID-19 должен был быть подтвержден комплексом факторов, включая повышение уровня HbA1с при госпитализации, гликемический профиль пациента при поступлении и в динамике (в том числе, после окончания терапии ГКС), а также наличие данного заболевания в анамнезе у части больных.

Всем участникам исследования проводилось профессиональное НМГ в слепом режиме с помощью системы постоянного мониторинга i-PRO-2, разработанной компанией Medtronic. По НМГ оценивали средние показатели гликемии (днем, ночью, за 24 ч, в 3-часовых интервалах в течение суток), продолжительность (% времени) сохранения гликемии в целевом диапазоне, выше и ниже него. При этом целевыми считали показатели гликемии в пределах 6–10 ммоль/л, которые признаются оптимальными для сочетания COVID-19 и СД отечественными и иностранными экспертами [19][26]. Гипогликемию 1 и 2-го уровня диагностировали в соответствии с международными рекомендациями [27]. Определяли наличие, время возникновения, продолжительность гипогликемии, оценивали процент времени сохранения гликемии в диапазоне <3,9 ммоль/л. Определяли вариабельность гликемии по коэффициентам вариации, стандартному отклонению и показателю средней амплитуды колебаний гликемии (meanamplitudeofglycemicexcursions, или MAGE).

Проводились измерения уровня глюкозы на стационарном анализаторе, кратность которых определялась конкретной клинической ситуацией. Уровень НbА1с определяли при поступлении пациента в стационар на приборе NycoCardReaderII.

## Статистический анализ

При статистической обработке применяли пакеты программ Statistica 8.0 и MedCalc. Для сравнения количественных данных в двух независимых выборках использовали критерий Манна-Уитни, качественных данных — Хи-квадрат и Фишера, для оценки корреляционных связей — критерий Спирмена. При описании выборок использовали среднее ± квадратическое отклонение (М±S). Различия считали достоверными при р≤0,05.

## Этическая экспертиза

Проведение исследования было одобрено ЛЭКГБУЗ НО «Городская клиническая больница №13 Автозаводского района города Нижнего Новгорода» 06 июня 2020 года (протокол № 07/20).

## РЕЗУЛЬТАТЫ

## Описание выборок

За время исследования в основную и контрольную группу было включено по 21 больному.

Больные основной (COVID-19 в сочетании с СД2) и контрольной групп (СД2) ожидаемо не различались по полу (мужчин — по 7 (33,3%) в каждой группе; р=1,00), возрасту (64,3±8,50 и 62,3±5,96 года; р=0,333), уровню НbА1с (9,8±2,09 и 9,6±1,82%; р=0,670), а также по индексу массы тела (30,7±5,15 и 29,2±5,83 кг/м2; р=0,131). В обеих группах оказалось одинаковое число пациентов со стажем СД более 5 лет (по 16 человек, или по 76,2%; р=1,00). Основная и контрольная группы не различались по частоте выявления таких осложнений СД, как нефропатия (10 (47,6%) и 11 (52,4%); р=0,762), ретинопатия (7 (33,3%) и 11 (52,4%); р=0,213) и полинейропатия (16 (76,2%) и 11 (52,4%); р=0,110). Также группы были сопоставимы по распространенности наиболее значимых коморбидных состояний, включая артериальную гипертензию (17 (80,95%) и 16 (76,2%); р=0,714), ишемическую болезнь сердца (по 7 (33,3%) человек;р=1,00), острое нарушение мозгового кровообращения в анамнезе (по 1 (4,8%) больному; р=1,00), хроническую обструктивную болезнь легких (по 1 (4,8%) пациенту; р=1,00), заболевания желудочно-кишечного тракта (10 (47,6%) и 8 (38,1%) соответственно; р=0,533). Обе группы были сопоставимы по характеру сахароснижающей терапии, проводимой до госпитализации; в том числе инсулинотерапию исходно получали 13 (61,9%) больных в основной группеи 16 (76,2%) — в контрольной (р=0,317).Все пациенты основной и контрольной группы во время проведения НМГ находились на инсулинотерапии; ее базис-болюсный вариант был использован у 18 человек в каждой группе (по 85,7%). Из них базис-болюсную инсулинотерапию по схеме «инсулин средней продолжительности действия (НПХ) 2–3 раза в день + инсулин короткого действия перед завтраком, обедом и ужином» получали 13 (61,9%) человек в основной группе и 12 (57,1%) — в контрольной (р=0,76). Базис-болюсный режим по схеме «аналог инсулина длительного действия 1–2 раза в день + аналог инсулина ультракороткого действия перед завтраком, обедом и ужином» был использован соответственно у 5 (23,8%) и 6 (28,6%) пациентов (р=0,73). Оставшиеся получали инсулин средней продолжительности действия двукратно в сочетании с коррекционным введением инсулина короткого действия при необходимости. Средние суточные дозировки базального инсулина на момент проведения НМГ в основной и в контрольной группах наблюдения составили соответственно 28,6±12,26 Ед и 29,8±14,06 Ед (р=0,83), инсулина короткого действия — 37,4±18,43 Ед и 34,8±18,61 Ед (р=0,66). Больные основной группы получали терапию COVID-19 согласно актуальным на момент госпитализации стандартам; в том числе, всем пациентам назначались ГКС (у 10 из них данная терапия к моменту НМГ уже завершилась).

Нежелательных явлений в ходе исследования у пациентов основной и контрольной группы не наблюдалось.

На втором этапе работы сравнили две подгруппы больных с COVID-19 и СД2: 1) пациенты, получавшие системные ГКС во время проведения НМГ, и 2) пациенты, которым НМГ проводилось после отмены ГКС.

Анализ клинического материала показал, что из 21 больного основной группы ГКС на момент проведения НМГ получали 11 человек (10 — дексаметазон и 1 — метилпреднизолон), тогда как оставшиеся 10 завершили гормональную терапию за 1–5 дней до старта НМГ. Учитывая разницу в фармакокинетике применявшихся ГКС, на данном этапе работы было решено не включать в статистический анализ показатели НМГ единственного пациента, получавшего метилпреднизолон.

В итоге больные были разделены на две подгруппы: обследованные с помощью НМГ: 1) во время лечения дексаметазоном либо 2) после его отмены, с численностью по 10 пациентов в каждой; день старта НМГ в подгруппах 1 и 2 соответствовал в среднем 1,4±0,70 и 13,1±5,85 суткам госпитализации. Все вошедшие в подгруппу 1 получали дексаметазон в дозе 8–16 мг/сут внутривенно, в утренние часы.

Особенности гликемического профиля у пациентов с COVID-19 и сопутствующим СД2, госпитализированных в общее инфекционное отделение.

Результаты сравнительного анализа показателей гликемии у больных с СД2 типа, имеющих и не имеющих COVID-19, представлены в табл. 1.

**Table:** Таблица 1. Показатели непрерывного мониторирования глюкозы у больных СД2 типа с и без COVID-19Table 1. Indicators of continuous glucose monitoring in patients with type 2 diabetes with and without COVID-19

Признак	СД и COVID-19n=21	СД без COVID-19n=21	р
Средняя гликемия днем ммоль/л	12,0±2,77	10,0±1,81	0,012
Средняя гликемия ночью ммоль/л	9,8±3,61	8,1±1,67	0,039
Средняя гликемия 24 ч ммоль/л	11,2±2,93	9,4±1,80	0,023
% времени в целевом диапазоне (6-10 ммоль/л)	32,7±20,40	48,0±15,60	0,026
% времени выше целевого диапазона (6-10 ммоль/л)	55,7±28,30	36,3±19,40	0,024
% времени ниже целевого диапазона (6-10 ммоль/л)	11,6±17,46	19,3±17,00	0,035
Станд. отклонение днем ммоль/л	3,4±1,30	3,0±1,01	0,32
Станд. отклонение ночью ммоль/л	2,8±2,63	2,2±0,72	0,80
Станд. отклонение 24 ч ммоль/л	3,4±1,05	2,9±0,92	0,24
Коэфф. вариабельности днем %	29,2±10,60	29,5±12,22	0,88
Коэфф. вариабельности ночью %	26,8±13,97	27,1±9,69	0,60
Коэфф. вариабельности 24 ч %	31,4±10,01	32,4±10,29	0,86
Индекс MAGE ммоль/л	7,8±2,72	7,0±2,08	0,47
Доля пациентов с эпизодами гипогликемий, абс/%	7 (33,3%)	8 (38,1%)	0,75
% времени ниже 3,9 ммоль/л	2,25±4,35	5,4±6,36	0,16

Основополагающим отличием больных с COVID-19 от контроля стала значимо большая гликемия в любое время суток, при существенном удлинении продолжительности ее пребывания в диапазоне выше целевого, за счет укорочения нахождения в целевом диапазоне, а также в интервале ниже целевых значений (p<0,05 по всем перечисленным параметрам).

Все показатели НМГ, характеризующие вариабельность гликемии, не показали межгрупповых различий (p>0,05 для стандартных отклонений и коэффициентов вариации днем, ночью и в течение суток, а также параметра MAGE).

Эпизоды гипогликемии выявлялись более чем у трети больных в каждой из групп наблюдения (р=0,75). Подробнее условия, способствовавшие развитию эпизодов гипогликемии на фоне сочетанной патологии, будут рассмотрены ниже.

## Факторы, взаимосвязанные с нарастанием гипергликемии у больных с сочетанием COVID-19 и СД2

Для выявления факторов, которые взаимосвязаны с нарастанием гипергликемии у больных с сочетанием COVID-19 и СД2, был проведен корреляционный анализ (табл. 2). Представленные в таблице данные подтверждают прямые корреляционные взаимосвязи между параметрами НМГ, характеризующими выраженность и стойкость гипергликемии, с одной стороны, и показателями тяжести COVID-19 и СД2, с другой стороны. Также обращают внимание прямые корреляционные взаимосвязи между степенью гипергликемии и проводимой терапией ГКС.

**Table:** Таблица 2. Статистически значимые корреляции некоторых показателей НМГ с клиническими характеристиками пациентов в группе больных с СД2 и COVID-19Table 2. Statistically significant correlations of some LMWH parameters with clinical characteristics of patients in the group of patients with DM2 and COVID-19

Показатель	R	p
% времени выше целевого диапазона 6-10 ммоль/л
Ожирение	0,48	0,029
% пораж. легких по КТ исходно	0,44	0,039
Сатурация О2 при поступлении	-0,48	0,029
HbA1c при поступлении	0,65	0,003
Прием дексаметазона	0,47	0,030
% времени в целевом диапазоне 6-10 ммоль/л
% пораж. легких по КТ исходно	-0,59	0,008
Сатурация О2 при поступлении	0,48	0,029
HbA1c при поступлении	-0,56	0,013
D-димер при поступлении	-0,51	0,021
Прием дексаметазона	-0,46	0,036
Средняя гликемия 24 ч
Ожирение	0,57	0,010
% пораж. легких по КТ исходно	0,53	0,018
HbA1c при поступлении	0,51	0,021
Прием дексаметазона	0,45	0,038

## Особенности гликемического профиля пациентов с COVID-19 и СД2, которым НМГ проводилась во время и после отмены терапии дексаметазоном

Далее были проанализированы особенности гликемического профиля пациентов с COVID-19 и СД2, которым НМГ проводилась во время и после отмены терапии дексаметазоном (табл. 3).

**Table:** Таблица 3. Показатели непрерывного мониторирования глюкозы у больных СД с COVID-19, получающих и завершивших терапию дексаметазономTable 3. Indicators of continuous glucose monitoring in diabetic patients with COVID-19 receiving and completing dexamethasone therapy

Признак	После терапии дексаметазономn=10	Во время терапии дексаметазономn=10	р
Средняя гликемия днем ммоль/л	10,3±2,28	13,3±2,65	0,017
Средняя гликемия ночью ммоль/л	7,8±2,14	11,2±4,17	0,034
Средняя гликемия 24 ч ммоль/л	9,4±2,08	12,5±3,03	0,019
% времени в целевом диапазоне (6-10 ммоль/л)	37,0±20,84	28,7±20,19	0,66
% времени выше целевого диапазона (6-10 ммоль/л)	41,7±27,71	65,8±26,44	0,085
% времени ниже целевого диапазона (6-10 ммоль/л)	21,6±21,38	5,3±10,15	0,053
Станд. отклонение 24 ч ммоль/л	3,2±1,19	3,4±0,96	0,54
Коэффициент вариабельности сутки, %	35,0±10,54	28,6±9,12	0,11
Индекс MAGE ммоль/л	7,8±2,85	7,6±2,75	1,0
Доля пациентов с гипогликемиями, абс./%	6 (60)	0 (0)	0,000
Ср. max гликемия ммоль/л	15,8±3,68	20,9±2,09	0,005
Ср. min гликемия ммоль/л	4,4±2,39	7,3±3,49	0,008

Пациенты, получавшие дексаметазон на момент НМГ, характеризовались большей гликемией в разное время суток, тенденциями к увеличению % времени ее нахождения выше целевого диапазона и к укорочению времени в интервале ниже оптимальных значений, при полном отсутствии зафиксированных эпизодов гипогликемии.

У пациентов, завершивших лечение дексаметазоном, сохранялись проявления гипергликемии (особенно в дневное время, когда средняя концентрация глюкозы выходила за верхнюю границу целевого диапазона). Однако выраженность гипергликемических нарушений по ряду показателей оказалась значимо меньшей, чем у лиц, получавших ГКС на момент НМГ. Кроме того, у 60% больных, завершивших лечение ГКС, выявлялись эпизоды гипогликемии по результатам НМГ, которые чаще всего не были диагностированы при рутинной контроле гликемии с помощью стационарного анализатора (имелся лишь один случай выявления легкой гипогликемии днем).

Особенности гликемических профилей в подгруппах больных с сочетанной патологией, получавших и завершивших лечение дексаметазоном, наглядно представлены на рисунке 1 (приводится оценка по средним уровням гликемии в 3-часовых интервалах на протяжении суток).

**Figure:**
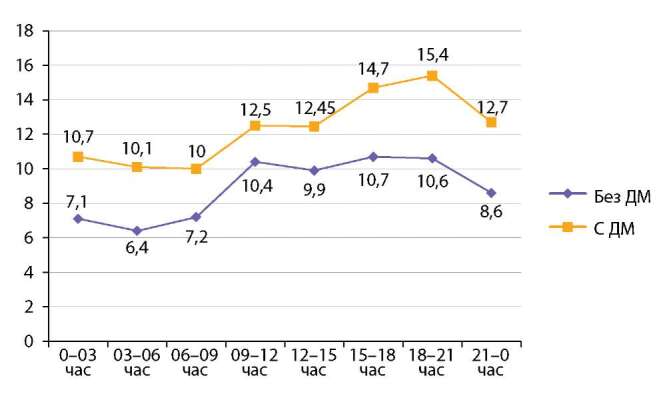
Рисунок 1. Показатели гликемии у больных с СД и COVID-19, у которых НМГ было проведено во время и после отмены лечения дексаметазоном (по средним уровням гликемии в 3-часовых интервалах на протяжении суток).Figure 1. Glycemic parameters in patients with DM and COVID-19 who underwent LMWH during and after discontinuation of dexamethasone treatment (mean glycemic levels at 3-hour intervals throughout the day). Примечание: p0–3 ч=0,004; p3–6 ч=0,001; p6–9 ч=0,012; p9–12 ч=0,123; p12–15 ч=0,063; p15–18 ч=0,007; p18–21ч=0,009; p21-0 ч=0,011. ДМ — дексаметазон

## Особенности и условия возникновения эпизодов гипогликемии у пациентов с сочетанием COVID-19 и СД2

Эпизоды гипогликемии по результатам НМГ были выявлены у 7 из 21 пациента с сочетанной патологией (33,3%), что составляет треть обследованных и говорит о достаточно широкой распространенности гипогликемии в основной группе. При этом 6 (28,6%) больных имели эпизоды гипогликемии 2 уровня, со снижением концентрации глюкозы ниже 3 ммоль/л (у 4 ее минимальный уровень достиг 2,8 ммоль/л, у 2 — 2,7 ммоль/л). Преобладающими были ночные эпизоды гипогликемии, которые выявлялись у 6 пациентов (28,6% всех лиц с сочетанием COVID-19 и СД2).

Среди пациентов, находившиеся на терапии дексаметазоном на момент НМГ, эпизодов гипогликемии зафиксировано не было. У пациентки, получавшей метилпреднизолон, имелось 2 дневных эпизода гипогликемии (один из них — 2 уровня). Таким образом, чаще всего гипогликемия возникала у пациентов, которые завершили лечение ГКС до старта НМГ.

## ОБСУЖДЕНИЕ

## Репрезентативность выборок

Набор участников проводился только в ГБУЗ НО «Городская клиническая больница №13 Автозаводского района города Нижнего Новгорода», что ограничивает репрезентативность полученной выборки.

## Сопоставление с другими публикациями

Больные основной и контрольной групп на момент поступления в стационар характеризовались одинаковой степенью декомпенсации СД, судя по значениям HbA1c (см. «Описание выборок»). Соответственно, более выраженная и стойкая гипергликемия в основной группе является изменением, которое обусловлено наличием COVID-19, и представляет собой характерную черту сочетанной патологии.

Даже в условиях стационара и проводимой инсулинотерапии, уровень гликемии у больных основной группы оставался в целевом диапазоне всего лишь на протяжении одной трети времени наблюдения (32,7±20,40%, см. табл. 1), что значительно ниже рекомендованных значений [28] и косвенно подтверждает сложность коррекции гипергликемии при СД2 и COVID-19, а также может способствовать ухудшению прогноза [14][15].

Высокая и стойкая гипергликемия у больных с сочетанием COVID-19 и СД2четко ассоциируется с худшей компенсацией СД на догоспитальном этапе (по HbA1c) и с большей тяжестью COVID-19 (по результатам КТ и показателям сатурации кислорода при поступлении, см. табл. 2).

Из применявшихся для лечения COVID-19 препаратов, только текущая терапия дексаметазоном обнаруживала достоверные корреляционные взаимосвязи с параметрами НМГ (прямые — со степенью гипергликемии и временем нахождения гликемии в диапазоне выше целевых значений, обратную — со временем ее пребывания в целевом интервале).

Нельзя исключить и роль ожирения в поддержании гипергликемии при COVID-19 м СД2, на что указывают прямые корреляционные взаимосвязи между его наличием и такими показателями НМГ, как средняя гликемия за 24 часа и процентвремени ее нахождения в диапазоне выше целевого.

Также обращает внимание обратная взаимосвязь доливремени нахождения гликемии в целевом диапазоне с уровнем D-димера в начале госпитализации, что может говорить об ассоциации дисгликемии с тромбогенными нарушениями и, возможно, об общности некоторых патогенетических звеньев формирования коагуляционных нарушений при COVID-19 и при СД2.

Несмотря на значимо большую выраженность и длительность гипергликемии, в основной группе имелась парадоксально высокая и близкая к контролю доля пациентов с эпизодами гипогликемии (33,3 vs 38,1%; р=0,75). Неожиданным было и отсутствие отличий от контроля по среднему проценту времени нахождения в зоне гипогликемии <3,9 ммоль/л (р=0,16) (невзирая на более короткое время пребывания гликемии ниже порога в 6 ммоль/л (р=0,035), и при сходной вариабельности (p>0,05 по всем параметрам, см. табл.1)).

Согласно дальнейшему анализу оказалась важной связь гипогликемии с особенностями проводимой терапии COVID-19 на момент НМГ, что, прежде всего, касается применения ГКС. В этом плане, основная группа больных с сочетанной патологией может быть условно подразделена на 3 подгруппы: 1) находившиеся на терапии дексаметазоном во время НМГ (n=10), 2) завершившие лечение дексаметазоном на момент проведения НМГ (n=10), 3) получающие метилпреднизолон в период исследования НМГ (n=1).

Как уже отмечалось, у пациентов, находившиеся на терапии дексаметазоном на момент НМГ, эпизодов гипогликемии зафиксировано не было. Основной проблемой с точки зрения гликемического контроля у них являлась выраженная склонность к гипергликемии, со значительным сокращением времени нахождения гликемии в целевом диапазоне. Все эти больные были недавно госпитализированными, клинически и лабораторно имели признаки острой воспалительной реакции (что предполагает выброс в кровь провоспалительных цитокинов и их негативное воздействие на чувствительность тканей к инсулину [1][3][5]). Вероятно, по этой причине, несмотря на многократный контроль гликемии в течение суток и, при необходимости, коррекцию инсулинотерапии, показатели НМГ у них оказались далеки от оптимальных.

У пациентов, завершивших лечение дексаметазоном, сохранялись проявления гипергликемии (особенно в дневное время, когда средняя концентрация глюкозы выходила за верхнюю границу целевого диапазона). Однако выраженность гипергликемических нарушений по ряду показателей оказалась значимо меньшей, чем у лиц, получавших ГКС на момент НМГ. Данные различия могут объясняться как снижением активности воспаления (отчасти, и за счет эффективного лечения COVID-19, включая предшествующее использование ГКС), так и изменениями терапии (окончание приема ГКС, оптимизация сахароснижающей терапии в ходе госпитализации). Наиболее же радикальным отличием подгруппы пациентов, завершивших лечение дексаметазоном, явилось именно наличие эпизодов гипогликемии, которые возникали у 6 (60%) больных. Следует уточнить, что в основной группе наблюдения (n=21) выявлялось 7 больных с гипогликемией; из них 6 завершили лечение дексаметазоном, а 1 получал метилпреднизолон на момент проведения НМГ (его данные на текущем этапе работы не учитывали).

Одним из факторов, способствующих гипогликемии после отмены ГКС, могло стать неадекватное снижение дозы инсулина. В соответствии с локальной клинической практикой, в течение трехдневного периода после отмены ГКС осуществлялся обязательный контроль гликемии натощак, до и после каждого приема пищи, а такжеперед сном. На основании полученного гликемического профиля в индивидуальном порядке проводилась титрация доз инсулина (как базального, так и короткого действия). С учетом наших данных, в указанный период времени была бы также целесообразной и оценка ночной гликемии, для оптимизации процесса титрации.

Согласно инструкции, дексаметазон является препаратом длительного действия, с продолжительностью биологического периода полувыведения до 36–54 ч. Считается, что его прямое воздействие на уровень гликемии может быть значимым в течение примерно 48 ч [3]. Этот факт отчасти объясняет стабильно большие показатели гликемии на протяжении суток в группе получавших дексаметазон; межгрупповые различия средних уровней гликемии утрачивали статистическую значимость только в интервалах 9–12 и 12–15 ч (что соответствовало времени очередной инфузии ГКС). При этом не следует забывать, что на снижение гликемии пациентов, завершивших лечение ГКС, безусловно, влияло не только отсутствие гипергликемического эффекта дексаметазона, но и достигнутое на фоне предшествующей терапии уменьшение активности воспаления, а также эффекты инсулинотерапии.

У пациентки, получавшей метилпреднизолон, имелось 2 дневных эпизода гипогликемии (один из них — 2 уровня), которые возникли в разные дни, но приблизительно в один временной период, примерно соответствующий окончанию действия препарата. В таблетированной форме метилпреднизолон имеет период полувыведения в диапазоне от 1,8 до 5,2 ч. При его приеме утром можно ожидать значительного снижения концентрации препарата в крови уже во второй половине дня (что и соответствовало времени появления зафиксированных у больной эпизодов гипогликемии). Хотя речь идет о единичном наблюдении, данная клиническая ситуация позволяет рекомендовать более частое исследование глюкозы крови у больных, получающих ГКС с небольшим периодом полувыведения; при этом конкретное время для контроля гликемии должно определяться с учетом фармакокинетики ГКС.

В подгруппе лиц, у которых НМГ была проведена после отмены дексаметазона, гипогликемия выявлялась у 6 человек из 10 (в 60% случаев), причем все эти больные имели ночные эпизоды гипогликемии, а половина из них — также и дневные. У большинства пациентов (5 из 6 с гипогликемией) она достигала 2 уровня. Также намного превышал рекомендованные значения и процент времени сохранения гипогликемических нарушений по НМГ (10,1±5,93%).

Полученные данные позволяют предположить, что у лиц с сочетанной патологией эпизоды гипогликемии, особенно ночной, являются серьезной проблемой, нередко возникающей в условиях отмены ГКС.

При этом в ходе ретроспективного сопоставления данных «слепого» НМГ с результатами рутинной оценки глюкозы крови стационарным анализатором было установлено, что чаще всего в реальной клинической практике эпизоды гипогликемии остаются не диагностированными (имелся лишь один случай выявления легкой гипогликемии днем). Следует отметить, что к моменту окончания терапии дексаметазоном, клиническое состояние всех больных с сочетанной патологией стабилизировалось. При этом рутинные исследования гликемии проводились реже и, как правило, выявляли лишь умеренную гипергликемию в пределах или выше целевого диапазона. В результате у лечащего врача не появлялось настороженности в отношении возможной гипогликемии. В том числе не проводился и контроль гликемии ночью. По-видимому, было бы целесообразно рекомендовать как минимум 5–6-кратное исследование уровня глюкозы крови (с обязательной ее оценкой в ночное время) даже стабильным больным с сочетанной патологией, на стационарном этапе, после окончания лечения дексаметазоном.

## Клиническая значимость результатов

Полученные в нашем исследовании результаты позволили уточнить особенности гликемического профиля пациентов с COVID-19 и СД2, получить новые данные о распространенности и характере дисгликемических нарушений во время и после окончания терапии ГКС у госпитализированных больных с сочетанной патологией.

## Ограничения исследования

Выборки пациентов были сформированы на базе одного центра, в связи с чем могут неполно отражать спектр и тяжесть дисгликемических нарушений у пациентов с COVID-19 и СД2, госпитализированных в общее отделение инфекционного госпиталя.

## Направления дальнейших исследований

В дальнейшем планируется проведение проспективного исследования на базе нескольких клинических центров для выявления предикторов риска серьезных дисгликемических нарушений (в том числе клинически значимых эпизодов гипогликемии) у госпитализированных больных с COVID-19 и СД2, а также для разработки мер по их профилактике.

## ЗАКЛЮЧЕНИЕ

Больные COVID-19 и СД2 отличаются более выраженной, устойчивой и трудно корректируемой гипергликемией, вследствие чего время нахождения уровня глюкозы в целевом диапазоне у них не превышает одной трети длительности НМГ.

Согласно корреляционному анализу, степень гипергликемии возрастает по мере утяжеления протекания COVID-19, при худшей компенсации СД на догоспитальном этапе, наличии ожирения, а также на фоне приема ГКС. Время нахождения гликемии в целевом диапазоне обратно связано с уровнем D-димера, что не исключает ассоциации дисгликемии с тромбогенными нарушениями.

У трети больных COVID-19 и СД2 имелись эпизоды гипогликемии. При этом более чем у четверти пациентов с сочетанной патологией они возникали в ночное время, были клинически значимыми и достигали второго уровня по данным НМГ.

На состояние гликемического контроля влиял статус пациента по отношению к приему ГКС. Лица, получавшие дексаметазон, были склонны к более выраженной гипергликемии, со значительным сокращением времени нахождения глюкозы в целевом диапазоне, но при отсутствии гипогликемии. У больных, которым НМГ проводилась после отмены дексаметазона, гипергликемия была менее выраженной, но у 60% из них выявлялись эпизоды гипогликемии, часто — ночные и клинически значимые.

В реальной клинической практике эпизоды гипогликемии, которые возникают у пациентов с сочетанной патологией после отмены и/или прекращения действия ГКС, часто остаются не диагностированными. Было бы целесообразно рекомендовать, как минимум, 5–6-кратное исследование уровня глюкозы крови (с обязательной ее оценкой в ночное время) даже стабильным больным с сочетанной патологией, на стационарном этапе, после окончания лечения длительно действующими ГКС.

## ДОПОЛНИТЕЛЬНАЯ ИНФОРМАЦИЯ

Источники финансирования. Работа выполнена по инициативе авторов без привлечения финансирования.

Конфликт интересов. Авторы декларируют отсутствие явных и потенциальных конфликтов интересов, связанных с содержанием настоящей статьи.

Участие авторов. Стронгин Л.Г. — концепция и дизайн исследования, сбор данных и интерпретация результатов, написание текста рукописи; Некрасова Т.А. — сбор данных, статистический расчет и интерпретация результатов, написание текста рукописи; Беликина Д.В. — сбор данных, статистический расчет и интерпретация результатов, написание текста рукописи; Корнева К.Г. — сбор данных, интерпретация результатов, написание текста рукописи; Петров А.В. — сбор данных, интерпретация результатов, написание текста рукописи. Все авторы одобрили финальную версию статьи перед публикацией, выразили согласие нести ответственность за все аспекты работы, подразумевающую надлежащее изучение и решение вопросов, связанных с точностью или добросовестностью любой части работы.
